# Comparative Study on Selected Insulating Materials for Industrial Piping

**DOI:** 10.3390/ma17071601

**Published:** 2024-03-31

**Authors:** Jan Porzuczek

**Affiliations:** Department of Thermal Processes, Air Protection and Waste Utilization, Faculty of Environmental Engineering and Energy, Cracow University of Technology, Warszawska 24, 31-155 Krakow, Poland; jan.porzuczek@pk.edu.pl

**Keywords:** pipe insulation, pipe lagging, ISO 8497, ASTM C335, thermal conductivity, mineral wool, polyethylene foam (PEF), expanded polystyrene (EPS), flexible elastomeric foam (FEF), polyurethane foam (PUR)

## Abstract

This paper describes the results of an experimental assessment of the thermal conductivity of pipe insulation. The need for reducing energy loss in industrial piping systems makes the availability of relevant and reliable insulation materials of special importance. Several specimens of pipe laggings, made of different materials, including mineral wool, polyethylene foam (PEF), expanded polystyrene (EPS), flexible elastomeric foam (FEF) and polyurethane foam (PUR), were tested in accordance with the European standard ISO 8497. The thermal conductivity of the materials was measured for a wide range of temperatures. The results were compared with the values reported in the technical specifications as well as with the literature data. The assessment of measurement uncertainty was also described. The results showed that, in a few cases, thermal conductivity turned out to be greater than that declared by the manufacturer by as much as over 10%.

## 1. Introduction

Thermal insulation is a material that reduces inadvisable heat flow from or into a certain area [1]. Even prehistoric people used insulation materials, originating from plant or animal tissues, to protect themselves from the cold [2]. However, modern insulation materials appeared at the beginning of the 20th century [3,4]. The intense growth of industrialisation in the 19th century accelerated the development of materials that were able to replace obsolete forms of insulation. A focus on energy conservation and therefore energy cost reduction obliged insulation manufacturers to provide improved but also cost-efficient materials. The sustainable development trend, which is still increasing nowadays, should motivate engineers to thoroughly consider the adopted solutions. On the other hand, rigorous expectations and high market competition may lead to the provision of materials that are not thoroughly validated.

Heat loss through the walls of industrial piping is considered to be one of the most significant factors driving the exploitation costs of technical installations [5]. It prompts an in-depth analysis and optimisation of insulation choices [6,7]. In industrial piping, insulation not only reduces heat loss but also ensures personnel safety. Other properties, such as noise reduction or fire resistance, might also be crucial in certain applications. In applications where moisture may penetrate the insulation, resistance to dampness is another factor to consider [8,9]. Therefore, there is no one material that is suitable for all installations—even if one might have better thermal properties, another can be more appropriate for the overall demands. Modern approaches to insulation layer optimisation should also take into account a lifecycle cost analysis (LCC) [10], not only the initial costs.

Nowadays, the most conventional materials used for piping insulation are as follows [1,4,11]:

- Mineral wool (MW);
- Glass wool (GW);
- Polyurethane foam (PUR);
- Polyisocyanurate foam (PIR);
- Expanded polystyrene (EPS);
- Extruded polystyrene (XPS);
- Polyethylene foam (PEF);
- Flexible elastomeric foam (FEF);
- Phenolic foam (PF);
- Foam glass (FG).

The comparisons of these materials themselves have been widely presented in the literature, e.g., in [12,13,14,15], mostly for flat slabs. It is noteworthy that there are virtually no comparisons for pipe insulation.

There are several classes of less commonly used materials reported in the literature. Most authors emphasise the need for increasing sustainability in the production of insulation materials. Therefore, materials that are based on renewable sources are the focus of scientific attention. One example might be geopolymers, which are a group of materials that are based on aluminosilicate [16]. Foamed forms of geopolymers have become a novel insulation material with valuable properties. Geopolymers can be also produced from waste material, e.g., fly ash from coal-fired power plants [17]. There are more examples of the use of waste materials to produce insulation. Paper or textile waste can be shredded and coated with reinforcement material to improve their resistance to fire, moisture or fungi [18]. On the other hand, sustainable insulation may be also made of more traditional materials, such as sheep wool, coconut or cotton fibres, jute, cork, etc., which have been used for several thousand years now [19,20]. Most of the above-mentioned materials may be used only in a form which is not suitable for pipe insulation (e.g., flat slab or as a blown-in insulation); thus, only conventional materials were chosen for experimental investigation in this research study.

As mentioned, there is a lot more research reported on flat forms of insulation than on circular ones [21]. However, the results acquired for flat insulation might not be adequate for materials in the form of lagging around a pipe. Materials formed into cylindrical shapes often have a different internal geometry, density distribution or cell shape [22]. Moreover, the thermal properties of the material significantly depend on the direction of heat flow. Commercially available insulation materials, in the form of laggings or shells, are characterised by a slightly larger internal diameter than the outside diameter of the pipe, which enables ease of application. This, in turn, creates an air gap of variable thickness between the pipe and the insulation. Some insulation products also have radial incisions to make the insulation more easily mounted onto a pipe. Consequently, the ‘apparent’ (or effective) thermal conductivity of a product may only be measured using pipe insulation testers. Natural convection around cylindrical insulation will cause a nonuniform surface temperature, which also cannot be replicated in a flat slab testing apparatus. Therefore, if the measured values are to be representative for end-use performance, the measurements ought to be taken using a pipe test apparatus rather than a heat flow meter or the guarded hot plate method [22]. In most cases, manufacturers do not inform whether their product’s thermal properties were determined for the insulation product (e.g., lagging or shell) including the air gap or for the insulation material itself. In this research study, all measurements were made for insulation mounted in the test apparatus in a way similar to how it is used in real installations and, therefore, included the air gap.

Since all commercially available pipe insulation materials are characterised by deviations in physical properties such as density, wall thickness, installation methods, etc., there is a corresponding variation in their thermal properties. In the European Union, the manufacturers of insulation materials may state conformity with the ISO 13787 standard [23], which regulates the method of declaring the thermal properties of their product. According to the standard, values of thermal conductivity and its temperature dependence should be declared in a tabular form or the form of the equation. The standard describes the procedure of selecting specimens for testing, the measurement methods and the procedure of validation of the declared values. Particularly, for each sample selected for testing, the measured thermal conductivity must not exceed the declared value by more than 10%. Otherwise, the result of the validation procedure is negative and the manufacturer should change the declared values. Therefore, none of the commercially available insulation materials can exceed a 10% limit above the declared value. Moreover, the manufacturer has to ensure that the declared thermal value includes the effects of ageing (corresponding to a reasonably expected service lifetime under normal conditions) and dispersion in the measured values. The research that involves the accelerated ageing procedure is time-consuming and, therefore, costly [24]. It is noteworthy that some of the manufacturers may declare only one value of thermal conductivity, for temperature 10 °C, in accordance with harmonised European standards (e.g., EN 13162 for mineral wool [25]). However, for application in a wide temperature range, applying a constant value of thermal properties is inadequate. For every material, its thermal conductivity increases with temperature; still, the rates of change are different. Furthermore, for some of the materials, the thermal conductivity increases approximately linear with temperature (within the considered temperature range), while, for the others, this relationship might be strongly nonlinear.

Among several parameters that are used to describe thermal properties of pipe insulations, the thermal conductivity (*λ*) is the most common. It can be calculated using Equation (1):

(1) $$\lambda =\frac{Q\ln\left(D_{2}/D_{0}\right)}{2\pi L(T_{0}-T_{2})},$$

where *Q* stands for the radial heat flux through the area of the test section of the length *L*, *D*_2_ and *D*_0_ are the external diameters of the insulation and the heating pipe, respectively, and *T*_2_ and *T*_0_ are the temperatures of relevant surfaces.

In this research, randomly selected specimens of different pipe insulation materials were tested at least four temperature points. The method according to the ISO 8497 [22] was adopted using the apparatus of the highest precision to verify how the measured thermal conductivity correlates with the declared value. This research was not meant to provide statistical data about thermal property distribution for different materials but rather its validation based on commercially available samples. According to the accepted standards, most of the specimens cannot exceed the declared values and none of them shall exceed the +10% limit. The need for a reduction in the energy loss in the industrial piping systems makes the availability of thoroughly validated insulation materials of special importance. As comparative study on modern pipe insulation based on its experimental investigation can hardly be found in the literature, the purpose of this paper is, therefore, to provide such results. In a broader context, it may also contribute to the discussion in the field of modern insulation quality and standardisation.

## 2. Materials and Methods

### 2.1. The Test Apparatus and the Method of Examination

The standard, yet the highest precision method of determining the thermal properties of pipe insulations in steady-state conditions, is the absolute method that utilises the apparatus of specified construction, according to ISO and ASTM standards [22,26]. To measure the thermal conductivity of a certain material, it is necessary to induce the radial heat flux through the material and measure the temperature difference between the external side of the insulation and the heater (in the steady state). For the insulation of determined geometry, thermal conductivity can be calculated from Equation (1). The principle of the absolute method of pipe insulation thermal conductivity measurement is therefore similar to the widely recognisable guarded heat plate (GHP) method [27]. The most significant difference is the geometry of the apparatus and the fact that, in pipe insulation testers, there is usually an air gap between the heater and the insulation.

In the ideal situation, no heat should flow axially; however, it is not possible to eliminate it in real apparatus. To minimise this unfavourable phenomenon, the apparatus is equipped with additional heaters at both ends of the heating pipe, which are controlled to reduce the temperature difference between the central part of the heating pipe and the endings. Moreover, the heating pipe has air gaps that also limit the axial heat flux.

The examination was prepared and conducted according to the European standard ISO 8497 [22], which is similar to the American standard ASTM C335 [26]. The ISO 8497 requires the use of at least four thermocouples on each surface to ensure the proper average temperature measurement. In this research, eight thermocouples (type T) were mounted onto each surface. The thermocouples were mounted on the heater and the external surface of the tested material using Kapton tape, which is a temperature-resistant material that provides stable adherence of the sensors to the examined surface. Thermocouple wires ought to be carried out on the surface (which is approximately isothermal) to avoid additional heat loss and, thus, local temperature drop.

The specimen’s outer diameter has to be thoroughly measured. The best precision may be obtained by measuring the circumference of the specimen using precision circometer tape and then calculating the diameter. The rest of the dimensions are as follows: the test section length and the outer diameter of the heating pipe are known from the manufacturer’s specification.

Before performing the thermal conductivity measurements, the investigated materials were dried in the laboratory drier at a temperature that did not exceed the maximum admissible temperature, until a constant mass was obtained. Then, the materials were conditioned in the standard atmosphere of the laboratory (temperature approximately 23 °C and relative humidity from 40% RH up to 65% RH) to reach equilibrium with the environment. This was achieved if two successive mass measurements within a 24 h interval did not differ by more than 0.5%. The specimens were also checked to determine whether any loss in their weight had occurred, which might have happened if the thermal decomposition of the tested material appeared during the investigation.

**Figure 1:**
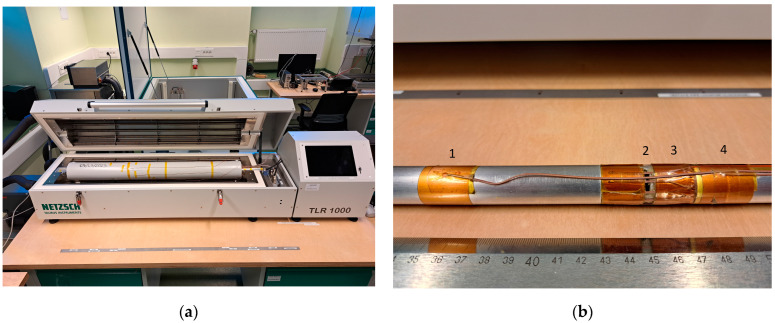
The test apparatus and the heating pipe: (**a**) Netzsch Taurus TLR 1000 apparatus; (**b**) heating pipe: (1) guarded end thermocouple, (2) the gap between test area and the guarded end, (3) thermoelectric chain, (4) one of the ‘hot side’ thermocouples.

The apparatus used in this research was Netzsch Taurus TLR 1000 (NETZSCH TAURUS Instruments GmbH, Weimar, Germany) (Figure 1) [28]. It allows measurements in a wide temperature range, nominally −15 ÷ 150 °C. Since the instrument is equipped with a heating/cooling jacket, it is possible to carry out an experiment that is independent of ambient temperature. The control system of the TLR 1000 gives the possibility to set the average temperature of the specimen as well as the temperature difference between the ‘hot’ and ‘cold’ sides. In this research, the temperature difference was set to 20K. Because the measurement method demands a steady-state heat transfer, it is controlled (via instrument software—Lambda 2012 Tube, ver. 12062016) where three consecutive measurement results (from 30 min. averaging each) do not vary more than 0.3%.

### 2.2. Specimens of Insulation Materials

**Figure 2:**
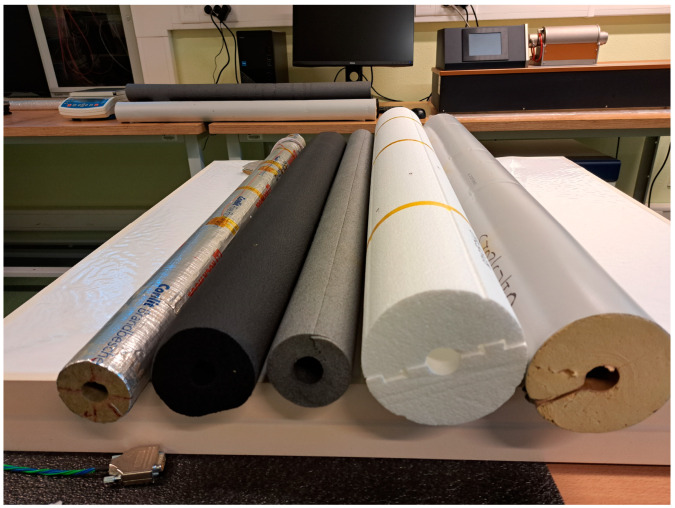
Examples of tested specimens: mineral wool (one of the reference specimens), FEF, PEF, EPS and PUR (from left to right).

For the experimental comparison of declared and measured values of thermal conductivity, 10 specimens were selected from the commercially available pipe insulations. The specimens are made of different conventional materials, such as mineral wool, polyurethane foam, polyethylene foam, FEF and expanded polystyrene. These materials were selected as they are very popular and widely available and, consequently, are common in most of applications, especially HVAC or refrigeration engineering. The basic properties of the specimens are juxtaposed in the table (Table 1). The specimens are characterised by different thicknesses; however, all of them are suitable to be mounted on a pipe of nominal 20 mm diameter; thus, the heating pipe of the same diameter was used during investigations. A few of the tested specimens are shown in the picture (Figure 2). It is worth noting that some of the manufacturers have declared temperature dependency of thermal conductivity in several points (or give a functional relationship), while the others declared only two or even one temperature point.

Since the certified reference materials (CRM) for the adopted method are not available, it was necessary to provide another method for verification of the test apparatus. Two of the specimens (proved as the most stable ones [29]) were agreed to be the reference materials (REF-1 and REF-2). To ensure the highest possible quality of the performed measurements, the reference materials were tested in other laboratories with accreditation in the scope of ISO 8497. Comparison of the values obtained for reference materials with approved laboratories gives a reliable assessment of measurement quality. Measurement uncertainty is discussed further in Section 2.3. The materials chosen to be the reference specimens were made of medium-density mineral wool with a reinforced aluminium coating. The thermal conductivity of the reference specimens covers most of the expected range of values—approx. 0.03 ÷ 0.05 W/(m × K).

The mineral wool specimens MW-1 and MW-2 were selected from one manufacturer; however, they differ in thickness. Similarly, for polyurethane foam specimens, two low-density PUR laggings of different thicknesses were chosen. Since the popularity of flexible elastomeric foams is still increasing, two specimens from different vendors were selected for the investigation. For PEF and EPS, one sample from each material was selected. As the EPS-1 specimen has only one *λ* declared (at 10 °C), values at other temperatures were calculated according to the research [12]. All the specimens were prepared for measurement as described in Section 2.1.

### 2.3. Evaluation of the Measurement Uncertainty

Every measurement can be performed with limited accuracy. Thus, the evaluation of the measurement uncertainty is an intrinsic part of the experimental investigation. According to the widely known guide [30], there is not only one correct method for uncertainty assessment. In the literature [31,32,33], one can find examples of different approaches to estimate the measurement uncertainty for the guarded heat plate method. There are no similar reports for pipe insulation testers.

Generally speaking, there are two ways for the evaluation of measurement uncertainty (or components of combined uncertainty) [30]:

- Type A—method of evaluation of uncertainty by the statistical analysis of a series of observations;
- Type B—method of evaluation of uncertainty by means other than the statistical analysis of a series of observations.

As the measurement of thermal properties of insulation materials in a steady state is a time-consuming procedure, there is virtually no possibility of repeating the measurement enough times to acquire a statistically significant number of repetitions. Consequently, type B is the most common approach in such measurements. It is worth noting that type B is not worse than type A; however, it might require a thorough investigation of the measurement method. To evaluate type B uncertainty, the study should take into consideration, e.g.:

- Data from apparatus calibration;
- Uncertainties assigned to reference materials;
- Manufacturer’s specifications;
- Knowledge and experience of researchers.

Since the guarded heat pipe measurement is the absolute method, the result of the measurement is biased by the following error components:

- Instrument error;
- Calibration correction;
- Repeatability error.

Having regard to all the components, the measurement equation can be written as follows (Equation (2)):

(2) $$\lambda =\lambda_{e}+\delta_{w}+\delta_{p}$$

where *λ* stands for the result of the measurement, *λ_e_* is readout from the apparatus (result estimation), *δ_w_* is calibration correction and *δ_p_* is repeatability error.

The best estimation of the result of the measurement is the readout from the apparatus. This readout is naturally biased by the instrument error and the uncertainty connected with this error can usually be found in the manufacturer’s specifications. Assuming that all the components of the apparatus error are within the specification limits, the overall apparatus uncertainty can be therefore evaluated. However, to ensure that any component of the instrument error does not exceed the accepted limit the apparatus should be regularly verified. According to Equation (1), the readout is calculated from:

- Geometric dimensions, which ought to be measured by the operator using validated instruments (e.g., rulers, circometers or callipers) and then entered to the apparatus software (Lambda 2012 Tube, ver. 12062016);
- Values which are measured by the apparatus itself: temperature and power delivered to the heater.

Thermocouples used for the temperature measurement should be calibrated in the calibration bath or dry-block calibrator. As the heating power is measured by the measurement of electric current and voltage, these quantities might be calibrated using a precision multimeter. The standard uncertainty of the verified instrument (*u_e_*) can be calculated using Equation (3). For the used apparatus, the expanded uncertainty *Uλ_e_,* according to the specification of the manufacturer [28], is 1.97%. Because this uncertainty in fact results from a few components (errors of measurement), it is characterised by normal probability distribution.

(3) $$u_{e}=\frac{U\lambda_{e}}{2},$$

To ensure the accuracy of the measurement, in most of the measurement methods, there are reference instruments or reference materials to compare with. For example, for the GHP method, the certified reference material (CRM) is available, e.g., [34], giving a possibility to achieve a lower measurement uncertainty. Such materials should be manufactured and tested in accordance with agreed standards, e.g., ISO 17034 [35]. That means the material has been proven to be highly stable and its certified properties were validated by several unrelated laboratories. In the case of measurement of the thermal conductivity of pipe insulation, there are no CRM available; thus, the accepted solution is to select the most stable specimens and verify their thermal properties during the interlaboratory comparison [21]. During apparatus calibration using reference material, a comparison between certified values and the instrument readout is made. In the conventional calibration procedure, the reference material is characterised by the properties known with significantly lower uncertainty than the calibrated instrument. After comparison, the corrections might be calculated and implemented in the measurement procedure. The problem is that the only reference material that is available for the pipe insulation testing method is a material examined by another laboratory. However, all the laboratories with accreditation for the measurement of thermal properties of pipe insulation are characterised by similar uncertainty. Consequently, it is not reasonable to adjust the obtained readout with the calibration correction. It is also prohibited if the measurement results ought to be reported in accordance with European standard EN 1946-5 [36]. For this reason, the expected value of the correction shall be zero with the expanded uncertainty $\left(U\delta_{w}\right)$ taken from the report of reference material investigation carried out by the other laboratory (3%). Assuming normal probability distribution, the standard uncertainty of calibration (*u_w_*) may be calculated from Equation (4).

(4) $$u_{w}=\frac{U\delta_{w}}{2},$$

The repeatability of the measurement (under repeatable conditions) gives crucial information about the impact of the random fluctuations on the measured value. The manufacturer of the apparatus declares the repeatability error (Δ$\delta_{p}$) to be less than 0.5%. During the investigation, this limit was verified. Contrary to the above-mentioned components, the probability propagation of uncertainty connected with repeatability is assumed to be square. Therefore, the standard uncertainty of repeatability (*u_p_*) can be calculated from Equation (5).

(5) $$u_{p}=\frac{\delta_{p}}{\sqrt{3}},$$

According to propagation of uncertainty, combined standard uncertainty (*u*) might be calculated from Equation (6).

(6) $$u=\sqrt{{u_{e}}^{2}+{u_{w}}^{2}+{u_{p}}^{2}},$$

The most common is to express the expanded uncertainty *U* with the coverage factor *k* = 2, which corresponds approximately to 95% coverage probability (Equation (7)).

(7) U=k·u

Summarising the assessment, the expanded uncertainty of measurement in this research is 3.64%. It is noteworthy that similar values are reported in the interlaboratory comparison report [21].

## 3. Results

The results are presented in graphs that show the comparison between declared and measured values for every specimen. As the measurements have been taken in four to five temperature points, the line of best fit was calculated and presented likewise. Where it was acceptable, the linear fit was adopted; otherwise, polynomial of second degree was used for a better fit. If the observed values exceed the declared ones, the +10% limit line is also presented in the graphs.

### 3.1. Reference Materials

**Figure 3:**
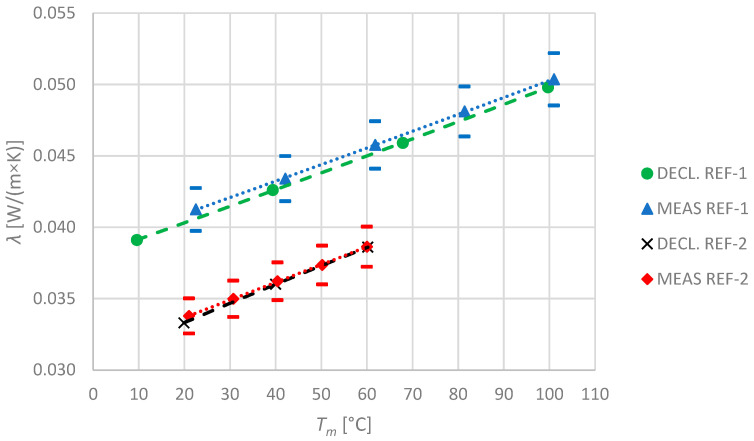
Reference and measured values of thermal conductivity for the specimens REF-1 and REF-2. The expanded measurement uncertainty is marked by hyphens.

A comparison of results, obtained in this research for the reference materials, with the ones provided by the other reliable laboratories is crucial as it proves the quality of measurements. The obtained results are shown in the Figure 3.

For REF-1, the maximum observed difference was approximately 1.38%, while the average difference was less than 1.24%. For REF-2, the results were even better: the maximum difference was 1.01% and average 0.52%. In the figure, the expanded uncertainty of the measurements is also indicated to illustrate the scale of observed differences in comparison with this uncertainty.

The investigations were independent and none of the values, reported by other laboratories, had an impact on the measurement results shown in this paper. Therefore, the results of reference material measurements have clearly shown that, even if minor differences were observed, they were negligible; thus, quality of the measurements in this research is confirmed. This also means that, if other accredited laboratories investigated our specimens, they would report results that are very close to those presented in this research.

### 3.2. Mineral Wool

**Figure 4:**
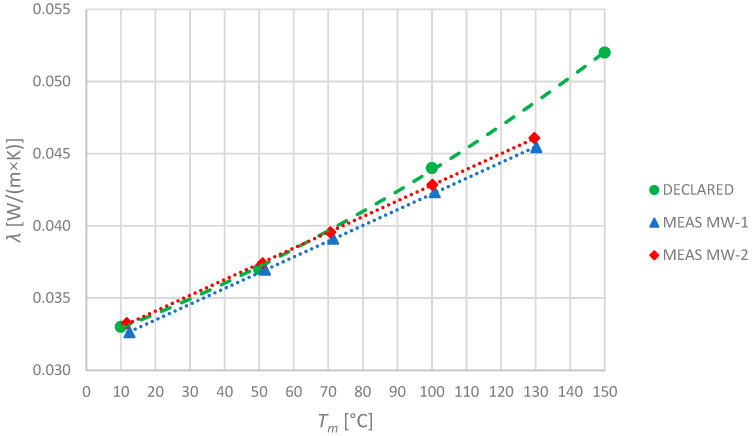
Declared and measured values of thermal conductivity for mineral wool specimens MW-1 and MW-2.

Since both specimens are made of the same material, their thermal properties should be similar. The obtained results are shown in Figure 4. The average difference between the specimens is approximately 1%. Taking into account the natural dispersion of parameters during production and the difference in thicknesses (which also has an impact on the measurement), the obtained results have shown good repeatability of manufacturing. All the observed results do not exceed the declared values in the test temperature range. The declared thermal conductivity of both the mineral wool specimens is nonlinear. One of the most important factors that has an impact on the linearity of this relationship is the thermal expansion of the material. This might be crucial at higher temperatures as the material can be used in temperatures up to 250 °C. In this research, the temperature range was limited to 130 °C and, because of the limited possibility of thermal expansion detection, this factor was not used to adjust the measured value. Consequently, the obtained relationships are approximately linear.

### 3.3. Polyurethane Foam

**Figure 5:**
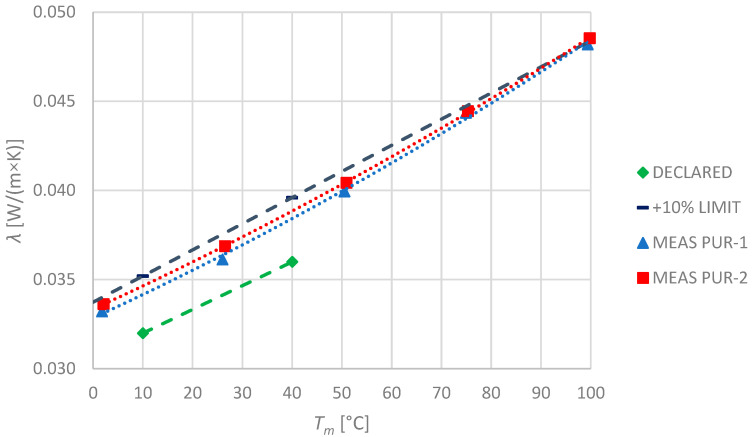
Declared and measured values of thermal conductivity for polyurethane foam specimens PUR-1 and PUR-2.

In the case of PUR specimens, the manufacturer has declared only two temperature points: 10 °C and 40 °C, for which the thermal conductivity shall not exceed 0.032 and 0.036 W/(m × K), respectively. Since this kind of product is suitable for use in temperatures up to 130 °C, producers’ declaration is incomprehensible. Observed values and their comparison with declared ones are shown in the picture (Figure 5). As the measured values exceed the manufacturer’s declaration, a +10% limit line is also presented in the graph.

Similarly to the mineral wool specimens, both of the PUR specimens were made of the same material; therefore, their thermal properties should be similar. They differ only in thickness. This study has confirmed that the thermal conductivity of both specimens differs approximately by 1.1%. Unfortunately, the obtained results exceed the declared one at mostly about 7–8%, reaching even 10% at 100 °C (for PUR-2). Polyurethane foam specimens are characterised by the lowest declared *λ*_10°C_ values (Table 1) over the rest. However, the investigation has shown significantly higher thermal conductivity that nonlinearly rises with temperature.

**Figure 6:**
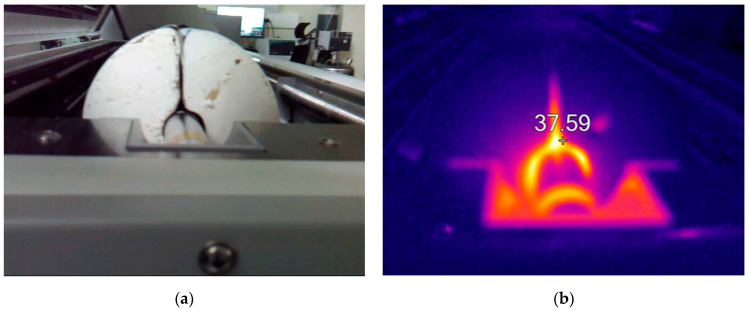
Polyurethane foam specimen—the radial air gap (**a**) PUR-1 specimen, the vertical position of the air gap; (**b**) thermal imaging of the fin-like action of the paper layer (‘+’ shows the highest temperature).

One of the reasons for this phenomenon might be that the internal layer, made of paper, that cuts through the insulation, acts similarly to a fin increasing the heat loss (Figure 6). Moreover, convection in the radial air gap also increases heat loss, especially at higher temperatures. Due to the stiffness of the material, the radial air gap cannot be removed completely by squeezing (which is possible, e.g., for mineral wool).

Measurements have been taken in two positions of the air gap: vertical and horizontal; however, observed values of thermal conductivity were similar. Observed differences were less than 0.5%. Thus, the orientation of the radial air gap seemed to be irrelevant during installation. The radial air gap was closed on the outer surface of the sample with adhesive tape, limiting heat loss. During measurement, it was observed that this tape tended to shrink at higher temperatures.

### 3.4. Polyethylene Foam

**Figure 7:**
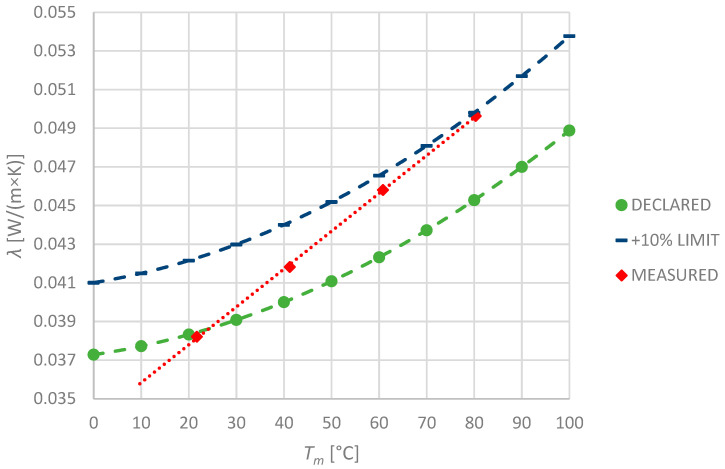
Declared and measured values of thermal conductivity for PEF-1 specimen.

Polyethylene-foam-based insulation is very popular nowadays for the low price and easiness of installation. The manufacturer of the specimen declared the thermal conductivity as the second-order polynomial; therefore, it is convenient to calculate the values of interest. Observed values and their comparison with declared ones are shown in the picture (Figure 7). As the measured values exceed the manufacturer’s declaration, the +10% limit line is also presented in the graph.

Even though the declared values are approximated by the nonlinear function of temperature, that nonlinearity has not occurred in measurements. It is noteworthy that the thermal conductivity of the specimen at 10 °C was much lower than the declared value. Nevertheless, above 24 °C, the measured thermal conductivity exceeds the declared value, reaching almost 10% at 80 °C.

In contrast to the other materials, the measurements of the polyethylene foam specimen were not repeatable. During five subsequent measurements, all of the observed values got significantly higher from one measurement to another. Exemplary *λ* values for 80 °C are shown in the table (Table 2). It is not clear whether this phenomenon is common for PEF insulation—it can be the aim of further investigation. Since the mass loss during the investigation was not detected, it is most likely that any thermal decomposition of the material has not occurred. However, internal changes in the material structure might have happened that could indicate that the declared temperature limit is too high for this material.

### 3.5. Flexible Elastomeric Foam

**Figure 8:**
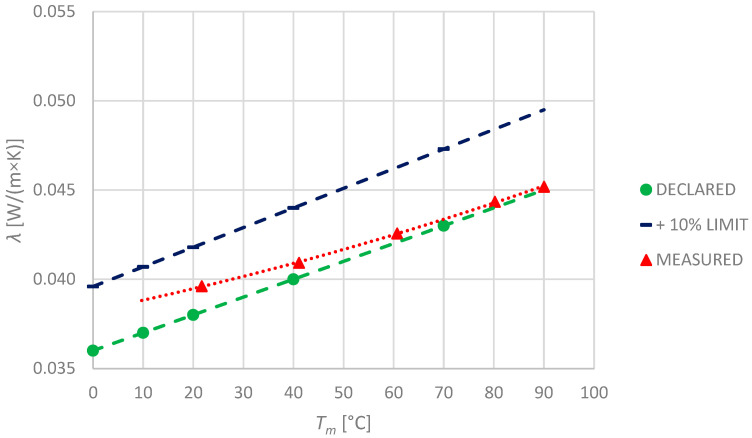
Declared and measured values of thermal conductivity for FEF-1 specimen.

**Figure 9:**
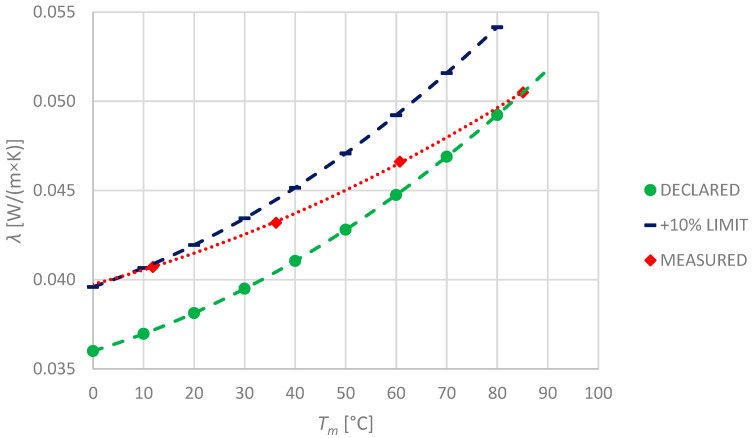
Declared and measured values of thermal conductivity for FEF-2 specimen.

Although the flexible elastomeric foam specimens FEF-1 and FEF-2 were made by different manufacturers, the declared properties of those materials are similar (Table 1). The noticeable difference is in the method of thermal conductivity declaration. The first of the manufacturers (FEF-1) declares thermal properties in seven temperature points and the relationship is approximately linear, while the second (FEF-2) states the relationship as the polynomial of second order. Observed values and their comparison with declared ones are shown in the pictures (Figure 8 and Figure 9). As the measured values exceed the manufacturers’ declaration, the +10% limit line is also presented in both graphs. Scales of axes of both graphs are set the same to illustrate the comparison.

Even though the specimens FEF-1 and FEF-2 are declared to have exactly the same thermal conductivity at 10 °C: 0.036 W/(m × K), for higher temperatures, the values diverge significantly. During the investigations, the results of measurement turned out to be greater than the declared ones for most of the temperature points. Only at the highest permissible temperature were the results close to the manufacturers’ declaration.

In contrast to other investigated materials, both elastomeric foam specimens turned out to be characterised by significant thermal expansion. Since thicknesses of the specimens increase with temperature, the correction needed to be implemented.

The difference between declared and measured values increases at lower temperatures. Despite the fact that this research does not cover measurement below 0 °C, it is clear from the observed trends that, in this condition, thermal conductivity of both analysed materials is significantly higher than declared. It is an important observation as the flexible elastomeric foam laggings are widely used in the field of refrigerating engineering to transport the media, such as coolants of very low temperatures (even −50 °C).

### 3.6. Expanded Polystyrene

**Figure 10:**
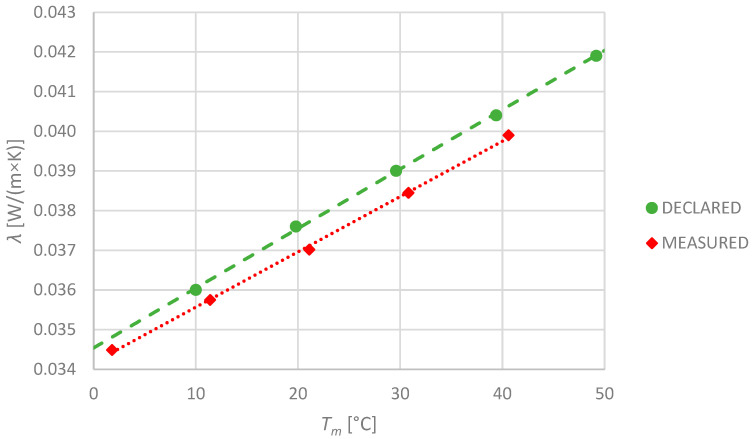
Declared and measured values of thermal conductivity for EPS-1 specimen.

Expanded polystyrene insulation is not very common in piping in contrast to, e.g., building wall insulation, where its position is well established. The manufacturer declared the thermal conductivity at 10 °C only: 0.036 W/(m × K). The other temperature points were calculated according to [12] and are illustrated in the picture (Figure 10). The results of the measurements are also presented in that graph. The measured values were lower than the declared ones of approximately 1.5% in the whole tested temperature range.

## 4. Discussion

In this research, in-depth analysis of the thermal performance of selected insulation materials has been carried out. The investigation method was used in accordance with the widely accepted European standard ISO 8497 and similar international standard ASTM C335. Since the high-precision apparatus was used during the measurements and the quality of the results was confirmed by the reference material measurements, the obtained results might be treated as reliable. For all of the specimens, the relationship between their thermal conductivity and the mean temperature was indicated. In the table (Table 3), the results are summarised (green colour represents the values which meet the specifications, while red colour represents the values which exceed the specifications). As it is most common to present the thermal conductivity at 10 °C, the juxtaposition is focused on these values. Thermal conductivity at 10 °C was calculated from the best-fit line. Measured values are rounded up and presented with a resolution of 0.001 W/(m × K), according to ISO 13787. Nevertheless, for any practical application, it is necessary to obtain the information of thermal conductivity for the whole applicable range, as it rises significantly with temperature.

Comparison of the obtained results with the declared values in the whole temperature range might be disturbing. Even though the vendors declare fulfilment of the requirements of the standards, the measurement results indicated that 50% of investigated specimens were characterised by significantly higher thermal conductivity *λ*_10°C_. Moreover, the thermal conductivity of two specimens exceeded even +10% limit. Naturally, there might be a few explanations for this observation. Quality control weakness during manufacturing might be only one of them. Underestimation of the dispersion of product parameters might also lead to providing products that are not thoroughly validated. It is, however, the least probable that the observed results were caused by specimen selection, as they were selected from different vendors, product lines and manufacturing batches. The probability that 50% of randomly selected specimens are defective is very low. However, in further research, the number of specimens should be increased. Since the amount of insulation material that is mounted on industrial piping or HVAC/refrigeration installations increases from one year to another, even minor exceeding might have a noticeable impact on overall heat loss and operational costs.

## 5. Conclusions

The tremendous impact that industrial installations have on total energy demand, and thus environmental and economic costs, should be the reason for a profound analysis of insulation intended to be applied. For this purpose, engineers, designers and end-users should be given reliable data that could be used for system optimisation or cost predictions. As was mentioned, it is disturbing that some of the investigated specimens were characterised by significantly higher thermal conductivity than that which they were declared. It should be mandatory for the manufacturers to provide information about thermal properties for the whole temperature range. It seems odd that material declared to be used up to, e.g., 130 °C (PUR) has thermal properties declared at 10 °C and 40 °C only. The usefulness of such information is questionable for most real applications. Moreover, the temporal stability of the material’s thermal properties should be given more attention. It can be assured only by comprehensive ageing tests that should be obligatory at least for new materials. It is certain that materials like PUR, PEF or FEF have a lot of advantages that, e.g., mineral wool does not, especially in the scope of weight or being moisture-proof. Nonetheless, a method for unequivocal declaration of the thermal properties seems to be demanded. It is also noteworthy that insulation documentation should contain information on whether the material was tested with the air gap between the heater and the insulation, as this provides information that is applicable to real conditions.

The side conclusion from this research is the need for CRM for pipe insulation testing. The use of certified material, which would be characterised by lower uncertainty than that used in this research, would decrease the total uncertainty of the measurements. This, in turn, might contribute to better quality control during production as well as more accurate measurements during laboratory tests.

## Figures and Tables

**Table 1:** List of the specimens of pipe laggings chosen for the comparison.

Material	Sample Name	*D*_2_*/D*_0_ (mm)	Density (kg/m^3^)	*λ_10°C_* (W/(m × K)) (Declared)	Max. Temp. (°C)	Method of *λ* Declaration
MW	REF-1	60/20	147	0.039	<100	4 ref. measurements
	REF-2	100/20	122	0.032	<60	3 ref. measurements
	MW-1	60/20	98.0	0.033	<250	4 points (10, 50, 100, 150 °C)
	MW-2	100/20	97.6	0.033	<250	4 points (10, 50, 100, 150 °C)
PUR	PUR-1	100/20	40.2	0.032	<130	2 points (10, 40 °C)
	PUR-2	70/20	43.0	0.032	<130	2 points (10, 40 °C)
PEF	PEF-1	70/20	19.7	0.038	<100	Equation (2nd polynomial)
FEF	FEF-1	70/20	62.0	0.037	<85	7 points (−30 ÷ 70 °C)
	FEF-2	84/20	59.4	0.037	<85	Equation (2nd polynomial)
EPS	EPS-1	120/20	16.5	0.036	<85	1 point (10 °C)

**Table 2:** Change in *λ* at 80 °C in five subsequent measurements for the PEF-1 specimen.

Measurement	1	2	3	4	5	DECL
*λ*_80°C_ (W/(m × K))	0.0496	0.0506	0.0512	0.0523	0.0527	0.0453
exceeding (%)	9.6	11.7	13.2	15.6	16.4	-

**Table 3:** Comparison of declared and measured *λ* at 10 °C (W/(m × K)) and validation of +10% permissible limit.

*λ* at 10 °C	MW-1	MW-2	PUR-1	PUR-2	PEF-1	FEF-1	FEF-2	EPS-1
Declared	0.033	0.033	0.032	0.032	0.038	0.037	0.037	0.036
Measured ^1^	0.033	0.033	0.034	0.035	0.036	0.039	0.041	0.036
+10% Valid. ^2^	Yes	Yes	Yes	No	No	Yes	Yes	Yes

^1^ Measured values are rounded up and presented with a resolution of 0.001 W/(m × K), according to ISO 13787. ^2^ In the whole measured temperature range.

## Data Availability

Data are contained within the article.
